# Consensus on Culturally Competent Mental Health Services Recommendations: A Modified Delphi Consensus Study With Ethnic Minorities, Carers, Public Advisors and Health Professionals

**DOI:** 10.1111/hex.70567

**Published:** 2026-02-18

**Authors:** Evgenia Stepanova, Sarah Croke, Maria Panagioti, Ge Yu, Yu Fu

**Affiliations:** ^1^ School of Pharmacy, Population Health Sciences Institute University of Newcastle Newcastle upon Tyne United Kingdom; ^2^ Division of Population Health, Health Services Research and Primary Care University of Manchester Manchester United Kingdom; ^3^ King's Health Economics, Health Services and Population Research Department, Institute of Psychiatry, Psychology & Neuroscience King's College London London United Kingdom; ^4^ Department of Primary Care and Mental Health University of Liverpool Liverpool United Kingdom

**Keywords:** co‐production, culturally competent, Delphi study, ethnic minority, mental health services

## Abstract

**Introduction:**

People from ethnic minorities in the UK face barriers to appropriate mental health care, including cultural stigma, language barriers and discriminatory practices. Culturally competent mental health services are proposed as a way to reduce these disparities, but implementation is limited.

**Aim:**

To establish consensus among ethnic minorities, carers, public advisors and health professionals on core components of culturally competent mental health services.

**Methods:**

A three‐stage Delphi process was conducted: two survey rounds followed by an online consensus workshop. Participants rated the importance of statements on a 9‐point Likert scale covering lived experience, systemic challenges, cultural sensitivity and community‐based support. Consensus was prespecified as ≥ 70% participants rating an item as important (scores of 7–9). Free‐text responses in Round 1 and workshop discussions informed wording and grouping of recommendations.

**Results:**

Sixty‐six took part in this study, including 34 service users, 3 carers, 3 public members and 26 professionals. Forty‐seven items reached consensus across four sections: lived experience of isolation and stigma living with mental health, systemic challenges for people from ethnic minorities seeking health services support, improving cultural sensitivity and support in services, and community‐based support. These were interpreted through four overarching themes in the workshop co‐production: understanding mental health in cultural context, raising awareness through community engagement, equipping interpreters and health professionals for inclusive mental health support, and driving systemic change towards collaboration, cultural sensitivity, and continuity of mental health care.

**Conclusions:**

This study co‐developed 46 recommendations that can be used as a checklist to support the development, design and delivery of culturally competent mental health services. The findings suggest that improving cultural competence will need system‐level and community‐informed changes, including stronger peer support, mental health education, advocacy and co‐production. The recommendations can guide commissioners and providers when reviewing service pathways and planning improvement work and offer a starting point for piloting and validating a practical checklist for routine use.

**Patient or Public Contribution:**

A project advisory group of four patient representatives and three team members met six times, reviewed and piloted survey materials, advised on recruitment, and contributed to analysis and final recommendations.

## 1 Background

Black, Asian, Mixed or other ethnic minority groups constitute 18% of the population in the United Kingdom, while the percentage of white British groups decreased by more than 6% between 2011 and 2021 [1]. This growing diversity presents significant challenges for health services due to differences in exposure to risk factors, patterns of mental health conditions and help‐seeking. For example, a higher prevalence of common mental health issues is reported amongst black and black British groups (23%) compared with white British groups (17%) [2]. These disparities were amplified during the COVID‐19 pandemic, which negatively affected mental health across the UK, with ethnic minorities experiencing a greater increase in psychological distress than while groups [3, 4]. The prevalence of psychological distress almost doubled among Asian groups during the first lockdown, rising from 18.7% to 34.9%, compared to a rise from 19.2% to 30.2% in white groups [5].

Despite inequality in prevalence, people from ethnic minority groups commonly report receiving less care than what they consider to be appropriate and face marked disparities in access, experience and outcomes [6]. Only 6% of Black adults receive mental health treatment, less than half compared to 13% in the white British population [2]. Recovery rates in the National Health Service (NHS) Talking Therapies also differ by ethnicity, with Asian or Asian British‐Pakistani males experiencing the lowest recovery rates at 33.5% while white Irish females have the highest recovery rates at 50.5%. Similarly, when examined by faith, patients identifying as Jain, Christian, and Jewish have the highest recovery rates, while Pagan and Muslim patients are among those with the lowest recovery rates [7]. People from ethnic minority communities are 40% more likely to access mental health services through the criminal justice system compared to their white counterparts [8], with Black individuals being more than three times more likely than white individuals to be detained under the Mental Health Act [9]. NHS England's Core20PLUS5 [10] framework identifies severe mental illness as a priority area for action on health inequalities, and the Patient and Carer Race Equality Framework (PCREF) [11] requires mental health providers to co‐produce and implement anti‐racist actions to reduce racial inequities in access, experience and outcomes.

Cultural competency in health services is commonly described as the integration of behaviours, attitudes, and policies that support effective engagement across diverse cultural contexts [12]. It involves principles and practices that promote respectful and meaningful interactions between systems, professionals, and people from different cultural or ethnic backgrounds, to improve both access to and the effectiveness of care for marginalised populations [13]. In mental health care, enhancing cultural competence has been proposed as one route to reducing disparities in access and service use [14, 15], but implementation has often focused on single outcomes, such as engagement in child and adolescent [16] services or recovery among Black and ethnic minority groups [17]. Recent evidence indicate that most interventions targeting mental health practitioners still focus on individual‐level knowledge, attitudes, and skills, with limited evidence on service‐level change or impact on inequalities in access and outcomes [18], despite increasing emphasis on moving beyond cultural competence training towards organisational accountability, system redesign, and equity‐oriented service transformation [11, 19].

The policy frameworks and research evidence underline a central conceptual and empirical uncertainty that there is still no agreed, service user‐informed operational definition of what culturally competent mental health services should look like in routine practice. Terms such as cultural competence, cultural humility and cultural safety are used in overlapping and inconsistent ways. Most available guidance emphasises individual knowledge and communication skills rather than clear, accountable service‐level components, pathways and partnerships with communities. Commissioners, providers and practitioners therefore lack a practical, consensus‐based checklist of core features to guide planning, implementation and evaluation of culturally competent mental health services.

This Delphi consensus study forms part of a wider mixed‐methods project [20, 21] on culturally competent mental health services in England, which also includes a literature review, analysis of electronic health records and qualitative interviews with ethnic minority service users. The Delphi work was designed to translate evidence and lived experience into a set of service‐level recommendations that reflect the priorities of ethnic minority service users, carers, public advisors and mental health professionals, and that can be used by policymakers, commissioners and practitioners to assess and strengthen cultural competence in mental health care.

## 2 Objective

This study aimed to establish consensus among ethnic minorities, carers and professionals on the core service‐level features of culturally competent mental health services, in order to inform commissioners, policymakers and practitioners.

## 3 Materials and Methods

### 3.1 Study Design

This study employed a modified Delphi process including the administration of two‐round online surveys and an online consensus workshop. A modified Delphi [22] process is widely used in healthcare settings to suit research needs and address potential limitations of the conventional technique [23]. The modification in the Delphi method took place during the first round, where an initial list of indicators was developed through a literature review [24] and qualitative interviews [25] with service users. The modified Delphi method was selected as it allowed expert groups to focus on the critical and most significant features of culturally competent mental health services identified by service users first‐hand. This was due to a lack of universal understanding and literature around the phenomenon [26]. We adopted an online stakeholder consensus meeting to allow a group of geographically diverse panellists to finalise the consensus.

The Delphi items were developed from a rapid review of changes in adult mental health services during COVID‐19 and from qualitative interviews with people from ethnic minority backgrounds, reported in detail elsewhere. The review aimed to describe how services adapted and to identify which changes improved or threatened access, continuity and equity, including for people from ethnic minority and other marginalised groups. It included 33 studies out of 6969 identified records from Cochrane CENTRAL, MEDLINE, Embase and PsycInfo. Search strategies combined controlled vocabulary and free‐text terms for three main concepts: adult mental health conditions and services, COVID‐19, and service delivery models. The qualitative work used semi‐structured interviews to explore how people from ethnic minority backgrounds understood mental health, experienced help‐seeking and contact with services, and what they thought culturally sensitive services should look like. A total of 32 adults with ethnic minority backgrounds were recruited through community and faith organisations, voluntary‐sector groups, social media and NHS mental health services. The topic guide covered experiences of distress and help‐seeking; experiences of NHS and voluntary‐sector support; cultural, religious, family and community influences; barriers and facilitators to engaging with services; and views on what culturally competent services should look like. Themes from the rapid review and qualitative study were translated into 81 candidate service‐level statements, which were then refined with the project advisory group to produce the 36 items used in Round 1 of the Delphi survey. Table 1 provides a summary of evidence from prior work informing the Delphi domains.

**Table 1:** Summary of evidence from prior work informing the Delphi domain.

Domain informing Delphi survey	Key findings from rapid review	Key findings from qualitative study	How this informed Delphi domains and items
Access, flexibility and continuity of care	Telehealth and new pathways often improved convenience and reduced missed appointments, but reduced hours and limited contact modes led to gaps in support and disrupted continuity for some groups.	Telehealth and new pathways often improved convenience and reduced missed appointments, but reduced hours and limited contact modes led to gaps in support and disrupted continuity for some groups.	Telehealth and new pathways often improved convenience and reduced missed appointments, but reduced hours and limited contact modes led to gaps in support and disrupted continuity for some groups.
Communication, language and information	Remote care created benefits for some but problems with privacy, technology and digital skills for others, including those at risk of digital exclusion.	Remote care created benefits for some but problems with privacy, technology and digital skills for others, including those at risk of digital exclusion.	Remote care created benefits for some but problems with privacy, technology and digital skills for others, including those at risk of digital exclusion.
Cultural stigma, safety and trust	Remote models could reduce anxiety for some but also made it harder to build rapport when privacy was limited or contact was brief.	Remote models could reduce anxiety for some but also made it harder to build rapport when privacy was limited or contact was brief.	Remote models could reduce anxiety for some, but also made it harder to build rapport when privacy was limited, or contact was brief.
Community and family support	When services were disrupted, informal and community support became more important; links between statutory and community provision were uneven.	When services were disrupted, informal and community support became more important; links between statutory and community provision were uneven.	When services were disrupted, informal and community support became more important; links between statutory and community provision were uneven.
Cultural tailoring of care and holistic support	Generic telehealth models were often rolled out quickly with little tailoring, raising concerns about widening inequalities for people with fewer resources or different expectations of care.	Generic telehealth models were often rolled out quickly with little tailoring, raising concerns about widening inequalities for people with fewer resources or different expectations of care.	Generic telehealth models were often rolled out quickly with little tailoring, raising concerns about widening inequalities for people with fewer resources or different expectations of care.
Staff training and service–community collaboration	Studies highlighted the need for guidance and training on safe and equitable remote care and on identifying who was being left behind.	Studies highlighted the need for guidance and training on safe and equitable remote care and on identifying who was being left behind.	Studies highlighted the need for guidance and training on safe and equitable remote care and on identifying who was being left behind.

All survey rounds were completed anonymously: participants were allocated unique identification numbers within the survey platform, and individual responses were not shared with the research team or other panellists. The study is reported in line with recommendations for the Conducting and REporting of DElphi Studies (CREDES) [27].

### 3.2 Expert Panel

The research evidence suggests that Delphi studies in mental health research often rely on professional expertise, excluding service users' experiences and voices [28]. To enable diversity in experiences and expertise, we targeted: people from ethnic minority groups with lived experiences of mental health problems, their families or carers, health professionals providing or planning mental health services, and public members who have relevant knowledge of mental health [29].

There are no established guidelines on the optimal Delphi study panel size, and the size of the panel members can vary from 10 to 1000^29^. A target of 24 to 60 panellists was set to ensure key stakeholders were sufficiently represented and the panel remained manageable, following a combination of purposive and snowball sampling frameworks to maximise heterogeneity. Adults able to use/access the Internet and email were eligible for inclusion if they met at least one of the following criteria: (1) person from an ethnic minority background with lived experience of using mental health services; (2) family member or carer of a person from an ethnic minority background living with mental health problems; (3) mental health professional providing services for people from ethnic minority backgrounds or with experience commissioning, planning or implementing mental health services; or (4) public advisor with an interest in mental health research or relevant knowledge or experience of mental health services.

People from ethnic minority groups, carers, and public members were recruited from local community organisations, and health professionals were from primary and secondary care settings, including general practitioner (GP) practices and disease‐specific services, with information widely shared via social media and professional networks by Mental Health Trusts and Integrated Care Boards in North England that provide support for ethnic minority communities.

### 3.3 Data Collection

#### 3.3.1 Round 1

Literature review [21] and qualitative interviews [30] by the study team informed a comprehensive list of criteria for achieving culturally competent mental health services, which were further reviewed by the advisory group to finalise the statements that seemed important to culturally competent mental health service provision. The OnlineSurveys platform [31] was used to distribute the survey. The survey was open for completion during 3 weeks (November 2023) with weekly reminders to maximise response rates. Before implementation, Round 1 was piloted by three study team members, two public advisors, one GP and one consultant psychiatrist, which led to minor structural changes for clarity and rewording for five items.

Each participant received an invitation email with a web link to the survey. The survey included a participant information sheet, a study consent, demographic questions (socio‐demographic and clinical characteristics for service users and carers and workplace, role and experience for professional panellists), and a list of statements on the features of culturally competent mental health services. Statements covered four sections focusing on: lived experience (7 items), systemic factors (16 items), delivery of mental health services (8 items) and ethnic minority communities (5 items). Completion of a round required panel members to rate each item in the questionnaire using a single, global 9‐point Likert scale indicating their agreement with the importance of the item, from ‘Not at all important’ to ‘important’ which was divided into three Sections 7–9 = “important”; 4–6 = “important, but not critical”; 1–3 = “not important.” Free‐text options were included at the end of each section to allow feedback and suggestions for additional items.

There is no single definition of consensus for Delphi studies [32]. Values between 51% and 80% of agreement have been commonly used as cut‐off points for consensus in the existing literature [28]. Therefore, we defined consensus as 70% rating an item with a score of 7 or above.

#### 3.3.2 Round 2

All participants who completed Round 1 were subsequently emailed links to Round 2 (January 2024). The same single 9‐point global importance scale was used in Round 2, again asking participants to judge overall importance. They were asked to rate the importance of items where consensus had not been achieved and the newly suggested items that arose from the free text responses in Round 1. They were also presented with results from items that had reached consensus for further modification.

Rating of each item from Round 1 was presented in an anonymous and aggregated form. For each item, participants were provided with summary statistics and four charts: the distribution of ratings for the whole panel, service users/carers, public advisors and professionals. Individual ratings were not identifiable. Participants were invited to reconsider their judgments and adjust their responses if they wished. No free text option was provided in Round 2 to minimise panellist and research burden.

Responses were analysed collectively for all participants, as well as separately for service users/carers, public advisors, and professionals. Items that reached consensus in Round 2 by all participants were included in the set of recommendations taken forward to the workshop. Panellist characteristics and importance ratings were descriptively analysed using Microsoft Excel.

#### 3.3.3 Consensus Workshop

Participants who had completed both rounds were invited to attend an online consensus workshop (March 2024) via a secure collaborative online meeting to facilitate access with participants over a large geographic area. Survey results and the list of items that had reached consensus were compiled into a workshop booklet, which was sent to participants in advance and shared on‐screen during the meeting. The 2‐h workshop followed a structured agenda led by two members of the research team, with one acting as facilitator and one as observer/note‐taker. Items were considered section by section. For each section, the facilitator briefly summarised the Round 1 and Round 2 ratings and then invited comments on areas of agreement or concern. A round‐robin format was used to ensure that service users, carers, public advisors and professionals all had opportunities to speak. Through open discussion, the group confirmed whether the 46 items that had reached quantitative consensus should be retained as recommendations and how they should be grouped for practical use. Where suggestions for rewording or combining items were made, the facilitator checked for explicit agreement from the group before noting changes. No additional voting took place during the workshop; instead, the workshop was used to confirm and interpret the consensus reached in the survey rounds and to co‐produce thematic groupings for the final recommendations.

### 3.4 Data Analysis

The demographic data were analysed using descriptive statistics. We summarised the distribution of participant characteristics (stakeholder group, gender, age and ethnicity) for Round 1 and Round 2 separately to describe attrition and to check for any obvious differences between completers and non‐completers. For each item, we calculated the proportion of participants rating it as important (scores 7–9), together with the range, median and interquartile range (IQR) of the 9‐point ratings across and within stakeholder panels.

The free‐text responses were analysed using directed content analysis [33]. Each of the four survey sections was treated as a main category, and each closed‐ended item was treated as a potential subcategory. ES and SC independently reviewed the free‐text data, coded comments into existing subcategories where they fit, and generated new subcategories where the content extended beyond the original items. Any discrepancies in coding were discussed and resolved between ES and SC, with further input from the wider research team where needed. New subcategories were then grouped into existing or new categories, and draft wording for new or revised statements was produced. These candidate items were reviewed by the project advisory group to check clarity, overlap and relevance, and only items judged to reflect distinct and important concepts were carried forward into the Round 2 questionnaire.

We also examined both consensus and stability in ratings across rounds. Consensus for an item was prespecified as at least 70% of panellists rating it as important (scores 7–9).31 Items that had met this threshold in Round 1 and were re‐presented in Round 2 were assessed for stability by comparing median scores and IQRs between rounds. Items were considered stable when median ratings remained unchanged or shifted by no more than one point on the 9‐point scale and when IQRs did not widen. Items that did not meet the consensus threshold but showed marked shifts in median ratings or narrowing IQRs were considered to be still evolving and were retained for re‐rating.

Workshop data were analysed using thematic analysis [34] to identify overarching themes and subthemes relating to mental health service provision. Field notes and transcript excerpts were read repeatedly, coded inductively and then organised into candidate themes. These themes were refined through discussion within the research team to ensure that they reflected the range of stakeholder perspectives and linked clearly to the final set of recommendations.

## 4 Results

A total of 74 panellists were invited to participate and were emailed the link to Round 1. Of these, 66 individuals completed Round 1, comprising 34 service users, 3 carers, 3 public advisors and 26 professionals, forming the expert panel. Fifty‐six (85%, 40 ethnic minorities and 16 professionals) completed Round 2, despite four reminders being sent. Attrition between Round 1 and Round 2 was 15% (10/66), and inspection of stakeholder group, gender, age and ethnicity indicated no notable differences in the distribution of characteristics between those who completed both rounds and those who only completed Round 1. Twelve participated in the consensus group workshop. Table 2 provides the characteristics of the expert panel.

**Table 2:** Characteristics of the expert panel in Round 1.

Type	Panellist	Round 1 (*n* = 66, 66/74 = 89%)
Ethnic minorities, *n* = 40	Role	
	Experience	
	Service users	34
	Family/carers	3
	Public advisors	3
	Gender	
	Female	17
	Male	20
	Transgender	3
	Age	
	18–25	0
	26–35	22
	36–45	4
	46–55	5
	56–65	3
	Ethnicity	
	Black, Black British, Black Welsh, Caribbean or African	19
	Asian, Asian British or Asian Welsh	11
	Mixed or Multiple ethnic groups	9
	Latin American	1
	Self‐reported mental health status (carers responding on behalf of the person they care for; 3 public advisors excluded)	
	Very good	6
	Good	5
	Changeable	17
	Poor	6
	Severe	3
Health professionals, *n* = 26	Professionals role	
	Primary care	5
	Psychology	4
	Psychiatry	11
	Community care	6
	Years of working	
	< 1	1
	1–2	2
	2–5	5
	5–10	4
	Over 10	14

**Figure 1:**
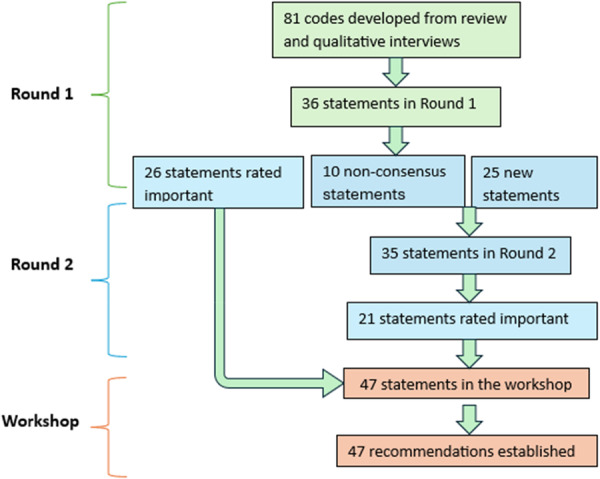
Delphi process.

The team compiled all the findings from the review and interviews into 81 codes. These statements were then independently reviewed by the project advisory group, who worked to refine them into clear and concise statements by grouping similar and repetitive ones. After a series of edits and four meetings with the project advisory group, 36 statements were finalised and included in Round 1. Across the two survey rounds, 46 items met the prespecified consensus threshold of at least 70% of participants rating the item as important (scores 7–9) and were taken forward to the consensus workshop (Figure 1).

### 4.1 Consensus Development

Of the 36 items included in Round 1, 25 items were rated as important, with a high degree of alignment between people from ethnic minorities and healthcare professionals. Seventeen people from ethnic minorities and nine healthcare professionals provided at least one free‐text response, resulting in 35 codes: 10 fitted within the pre‐specified subcategories and 25 formed new subcategories that were included as additional items in Round 2. In addition to the 10 items that did not reach consensus in Round 1, 35 items were surveyed in Round 2. Twenty‐one of these items met the consensus threshold. Overall, 46 items (25 from Round 1 and 21 from Round 2) were included in the final set of recommendations for the workshop without further amendments (Figure 1).

#### 4.1.1 Lived Experience of Isolation and Stigma in Mental Health Across Communities

Nine items on lived experience, isolation and stigma reached consensus as key components of culturally competent mental health services (Table 3). These items described mental illness as deeply isolating, highlighted the role of stigma within some ethnic minority communities, and stressed the importance of meaningful activity in protecting mental health.

**Table 3:** Consensus rating of lived experience of isolation and stigma in mental health across communities.

Description of item	% of important rating (scores 7–9 ≥ 70% on the 9‐point Likert scale)	
	Round 1 (*n* = 66)	Round 2 (*n* = 56)
Having a mental health problem feels isolating and lonely	85%	
Ethnic minority communities find it difficult to accept or understand what mental illness is, or what mental health is	79%	
It is very difficult to speak out about mental health problems (in community or to family)	88%	
There is much stigma about mental health in ethnic minority communities	88%	
Substance abuse as a means of hiding from mental health problem		70%
Participating in work or meaningful activities prevents or reduces feelings of anxiety or depression (e.g., voluntary work, training, etc.)		80%
Features of mental health affect people belonging to different ethnic or religious communities in different ways		88%
Different groups can find it easier or more difficult to accept mental health difficulties and to communicate what mental health really is		88%
A lack of understanding by most of society makes people with mental health difficulties feel isolated or confused		84%

Seven items were included in Round 1 under this section, and 10 were surveyed in Round 2, with seven additional items added from Round 1 free‐text. Four were rated important in Round 1 and five in Round 2, resulting in a total of nine items included in the final set of recommendations following the consensus workshop (Table 3).

#### 4.1.2 Systemic Challenges for Ethnic Minorities Seeking Mental Health Services

Twenty‐two items describing systemic challenges for ethnic minorities seeking mental health services reached consensus (Table 4). These items emphasised long and complex care pathways, fear and powerlessness when accessing services, misdiagnosis, and structural discrimination, as well as the importance of continuity, follow‐up, and training for frontline staff.

**Table 4:** Consensus rating of systemic challenges for ethnic minorities seeking mental health services.

Description of item	% of important rating (scores 7–9 ≥ 70% on the 9‐point Likert scale)	
	Round 1 (*n* = 66)	Round 2 (*n* = 56)
People from ethnic minorities spend far too long going through UK systems and/or mental health processes	70%	
Different cultures in the UK have different health and life expectations from each other (e.g. family ties, role of spirituality, traditions)	80%	
People from ethnic minorities feel powerless within health services when seeking mental health support	77%	
Accessing health services for mental health support can feel scary for people from ethnic minorities	82%	
People from ethnic minorities often need extra time with a health professional	70%	
People from ethnic minorities experience additional judgement and discrimination within the health and care services due to their cultural or religious needs	76%	
Language barriers between people from ethnic minorities and health professionals include not understanding cultural expressions or traditions	83%	
Ethnic minority people often get passed around because services are very disconnected	74%	
GPs and receptionists should have cultural training	83%	
All GPs, receptionists and health care staff should have training relating to mental health	89%	
Relationships between a service user and a health professional should be close, trusting and bonding	89%	
A lack of appropriate support or care can increase suicidal thoughts	91%	
Mental health services should provide support and work together with ethnic minority people, and their family members, in their community	92%	
There should be follow up and regular checks on ethnic minority people with mental health needs	88%	
It can be very hard to get a correct diagnosis for ethnic minority people		70%
Cultural observations can sometimes be mistaken for positive/negative symptoms of mental health issues by health professionals		70%
Not being able to access health professionals from the same or similar ethnic backgrounds is an additional barrier to people from ethnic minority backgrounds		84%
Mental health teams should have a lower threshold for accepting people into services sooner		71%
Mental health practitioners should be trained to engage patients around their culture and religion and accept racism can be a traumatic experience		93%
There is a severe need for more interpreters who can help with translations		79%
Many people do not have a laptop or do not know how to join Teams or Zoom meeting		73%
The communication barriers to accessing support for mental health may be quite different for people from ethnic minorities than from other groups		82%

Sixteen items were included in Round 1 under this section, and 12 were surveyed in Round 2, with eight additional items added from Round 1 free‐text. Fourteen were rated important in Round 1 and eight in Round 2, resulting in a total of 22 items included in the final set of recommendations following the consensus workshop (Table 4).

#### 4.1.3 Improving Cultural Sensitivity and Support in Mental Health Care

Eight items focused on improving cultural sensitivity and support within mental health services reached consensus (Table 5). These items highlighted the need for culturally appropriate care, recognition of overlapping marginalised identities, emotional intelligence among practitioners, and explicit training in cultural and religious sensitivity.

**Table 5:** Consensus rating of improving cultural sensitivity and support in mental health care.

Description of item	% of important rating (scores 7–9 ≥ 70% on the 9‐point Likert scale)	
	Round 1 (*n* = 66)	Round 2 (*n* = 56)
Social prescribing is a great tool for looking after mental health	74%	
It is a burden/exhausting to constantly have to explain cultural matters and backgrounds to mental health professionals	80%	
Mental health support should be culturally appropriate	91%	
Mental health support needs to consider the overlap of mental health with other highly stigmatised groups (e.g., substance use, LGBTQ+)		80%
Mental health services need to make sure people from ethnic minorities do not get lost in the system		91%
It is important that mental health practitioners have the emotional intelligence for the job		91%
Mental health professionals should be trained to be culturally sensitive, regardless of their own background		91%
Mental health services and hospitals should be aware and make allowances for people who refuse to take medications if it conflicts with their cultural or religious beliefs		77%

Eight items were included in Round 1 under this section, and 11 were surveyed in Round 2, with eight additional items added from Round 1 free‐text. Three were rated important in Round 1 and five in Round 2, resulting in a total of eight items included in the final set of recommendations following the consensus workshop (Table 5).

#### 4.1.4 Community‐Based Mental Health Support

Seven items on community‐based support reached consensus, underlining the importance of non‐clinical community resources, advocacy, peer support and sustained investment in community groups (Table 6). These items stressed that support offered within people's own communities, including faith and family networks, is integral to culturally competent care.

**Table 6:** Consensus rating of community‐based mental health support.

**Description of item**	% of important rating (scores 7–9 ≥ 70% on the 9‐point Likert scale)	
	Round 1 (*n* = 66)	Round 2 (*n* = 56)
Connecting ethnic minority service users to non‐clinical services in the community should be part of any treatment plan	82%	
An advocate who knows the cultural needs and the UK system can reduce fear and help with understanding the mental health journey	91%	
Charities/community groups give great support by engaging with ethnic minority service users as ‘people’ not ‘patients’	79%	
Peer training and peer support groups within ethnic minority communities should be part of mental health services	91%	
There is shortage of funding to support community groups for ethnic minority people with mental health problems	91%	
It is helpful and important to see support in the community talking to your family members		77%
Socialising and Faith in positive things can help to manage mental health issues better		77%

Five items were included in Round 1 under this section, and two were surveyed in Round 2, with both added from Round 1 free‐text. Five were rated important in Round 1 and two in Round 2, resulting in a total of seven items included in the final set of recommendations following the consensus workshop (Table 6).

### 4.2 Consensus Workshop

Twelve participants, including 7 service users, 2 carers, 1 public advisor and 2 professionals, attended the 2‐h online workshop. Participants were presented with the results from both rounds and then took part in a structured group discussion to explore areas of agreement and disagreement. The workshop confirmed the 46 consensus items and grouped them into four overarching themes that described how mental health services need to change: understanding mental health through a cultural lens; raising mental health awareness through multi‐faceted community engagement; equipping translators and health professionals for inclusive mental health support; and systemic change towards collaboration, cultural sensitivity, and continuity of mental health care. Overall, participants described how current services often feel fragmented, culturally insensitive and difficult to navigate, and how stronger relationships, community partnerships and culturally informed practice are needed to build trust and improve continuity of support.

#### 4.2.1 Understanding Mental Health Through a Cultural Lens

People from ethnic minorities described mental health as a contested and often stigmatised concept within some cultural and religious communities. They explained that mental distress was not always understood in clinical terms and was sometimes attributed to spiritual or moral causes. Consequently, seeking professional help could carry significant stigma, leading to shame, social exclusion, or silence within families.

Service users felt that these cultural perspectives were not always acknowledged by providers and called for greater cultural awareness and sensitivity to how mental health should be discussed and addressed. They emphasised the need for services to engage with communities in ways that recognised diverse religious or cultural beliefs and reduced stigma, including working with faith leaders and using language that aligned with community values.

#### 4.2.2 Raising Mental Health Awareness Through Multi‐Faceted Community Engagement

Participants expressed a strong desire for increased mental health awareness within ethnic minority communities. They felt that current efforts were limited and often failed to reach those most in need. Many emphasised the importance of using varied, culturally appropriate methods, for example, community events, religious gatherings, and social media to engage different groups.

Service users highlighted that awareness should go beyond information‐sharing and include open conversations that challenge stigma, normalise help‐seeking, and reflect the lived experiences of diverse communities. They called on service providers to work in partnership with community leaders and organisations to deliver mental health education in accessible and meaningful ways.

#### 4.2.3 Equipping Translators and Health Professionals for Inclusive Mental Health Support

Participants emphasised the need for both translators and health professionals to receive targeted training to improve the inclusivity and effectiveness of mental health care. They described instances where translators lacked an understanding of mental health terminology or concepts, which led to miscommunication and distress during consultations.

Service users also highlighted the importance of cultural and religious sensitivity among health professionals. They felt that a lack of awareness about cultural values, religious practices, and community norms often resulted in misunderstandings or care that did not feel respectful or relevant. Participants called for training that would enable both translators and clinicians to engage more empathetically and appropriately with diverse ethnic minority populations.

#### 4.2.4 Systemic Change Towards Collaboration, Cultural Sensitivity, and Continuity of Mental Health Care

Participants called for fundamental changes in how mental health services are structured and delivered. They highlighted the need for stronger collaboration between service users, health professionals, and service providers to ensure that care was responsive, respectful, and informed by lived experience.

Many service users described services as fragmented, culturally insensitive, and lacking in follow‐up support. They emphasised the importance of ongoing, relationship‐based care, where providers understood the cultural and religious contexts of service users' lives. Participants advocated for systemic improvements that prioritised cultural competence, built trust, and ensured continuity of care across different points of contact within the mental health system.

## 5 Discussion

This modified Delphi study brought together ethnic minority service users, carers, public advisors and professionals to agree a set of service‐level features that they regard as central to culturally competent mental health care. By grounding the Delphi instrument in a rapid review and qualitative interviews, the study moves beyond high‐level statements about cultural competence and generates a concrete, user‐informed checklist that reflects both individual experiences and system constraints. The findings point to cultural competence as a property of whole service systems rather than only of individual practitioners and link this explicitly to current policy ambitions to reduce racial and ethnic inequalities in mental health care.

A first key contribution is the way the study reinterprets isolation and stigma. Consistent with earlier work, participants described cultural stigma, silence and contested views of mental illness as powerful barriers to help‐seeking among ethnic minority groups [35, 36, 37]. The qualitative work and Delphi findings taken together show that distress is often framed in moral or spiritual terms, which can make biomedical explanations feel alien or unacceptable. This helps to explain why some service users experience contact with mental health services as shaming or invalidating rather than supportive. In this context, isolation is not just a symptom of illness but also an outcome of culturally unsafe care, where services neither acknowledge nor work with community‐held understandings of mental health [38]. This has implications for how services approach early intervention, with a need to engage with community and faith‐based frameworks rather than treating them simply as sources of “misunderstanding”.

The consensus items and workshop themes on community engagement highlight that raising mental health awareness is a long‐term, relational process, not a single intervention. Participants described current awareness efforts as limited, poorly targeted and often disconnected from the settings where people actually seek advice, such as religious spaces or informal peer networks. They called for education and discussion that confront stigma directly, use familiar language and stories, and are delivered in partnership with trusted community leaders. This aligns with evidence that community‐level and faith‐based interventions can improve mental health literacy and reduce stigma in Black and other racialised communities [39, 40, 41]. The present study adds by showing that service users regard such work as integral to culturally competent services, rather than as optional outreach. It also suggests that measures of cultural competence should include how services support, resource and learn from community partners, not only what happens within clinics.

Communication and language support formed a third major area of consensus. Participants identified multiple points at which communication breaks down: lack of clear written information, reliance on technical language, limited use of interpreters, and interpreters without mental health training. This echoes previous concerns about miscommunication in triadic consultations and the emotional load placed on interpreters [42, 43, 44]. The strong support for joint training of interpreters and clinicians, longer interpreted appointments, and supervision for interpreters indicates that translation is not simply a technical task but part of the therapeutic process. The findings therefore support a shift from seeing interpreters as a neutral “tool” towards understanding them as co‐workers who need preparation, support and involvement in service design. They also reinforce calls for training in cultural humility, structural factors and reflexive practice, and show how these ideas can be translated into specific service policies, such as supervision structures and time allocation [45].

The component on systemic challenges and continuity of care underlines the limits of focusing only on front‐line interactions. Participants described services as fragmented, difficult to navigate and reactive, with little planned follow‐up and weak links between primary care, specialist services and community organisations. Many of the Delphi items and workshop themes point to structural discrimination and the sense of “not being a priority”, which is consistent with broader evidence on structural inequities in mental health systems. These findings suggest that cultural competence cannot be achieved solely by improving communication within individual consultations. Instead, it requires changes to referral pathways, gatekeeping processes, follow‐up protocols and accountability mechanisms, so that people from ethnic minority backgrounds do not have to rely on crisis care or informal networks to fill gaps in support.

The study also has implications for how cultural competence is conceptualised in policy and practice. Current frameworks, e.g. Core20PLUS5 and the PCREF call for reductions in inequalities and for services to demonstrate anti‐racist, culturally competent practice, but offer limited operational detail on what this should look like at service level. The 46‐item checklist developed here provides a structured way for commissioners, providers and clinical teams to review their services against user‐informed criteria, covering access, communication, relationships, community partnership and continuity. It can be used to support equity audits, service specifications, quality improvement projects and co‐production with community partners, and can help local systems to identify where efforts and investment are most needed.

### 5.1 Limitations

There were fewer healthcare professionals than people from ethnic minorities involved, which may lead to underrepresented ratings from professionals. However, ratings between them were well aligned across groups. The online format may have excluded those with limited digital access or literacy. Future work should continue to prioritise inclusive engagement strategies to reach underserved populations.

## 6 Conclusion

This study co‐developed 46 recommendations that summarise what ethnic minority services, carers, public advisors and professionals regard as core features of culturally competent mental health services. The recommendations bring together lived experience and professional perspectives in a single, practical checklist that can be used to reflect on how far existing services align with culturally competent practice.

The checklist can support commissioners, providers and clinical teams to review local pathways, identify gaps, and plan targeted improvements in partnership with communities. It can inform service specifications, equity audits, staff training priorities and quality‐improvement work, and can provide a shared reference point for discussions between services and community organisations about what “good” looks like. The findings suggest the need for system‐level and community‐informed changes, rather than stand‐alone training, if services are to become more culturally competent. These changes are likely to include stronger collaboration with community and faith groups, better support for interpreters and front‐line staff, and more consistent follow‐up and continuity of care.

Next steps include piloting the checklist in different NHS and community mental health settings, assessing its acceptability and feasibility, and testing how well it captures variation in practice. Further work should examine the reliability and validity of the checklist and explore associations between checklist scores and service outcomes such as access, experience and inequalities. This will help to refine the tool and inform the design and evaluation of future interventions that aim to make mental health services more culturally competent and equitable.

## Author Contributions

**Evgenia Stepanova:** validation, formal analysis, project administration, writing – review and editing, writing – original draft, data curation, software. **Sarah Croke:** validation, formal analysis, project administration, writing – review and editing, writing – original draft, data curation, software. **Maria Panagioti:** writing – review and editing, validation, investigation, supervision, methodology. **Ge Yu:** writing – review and editing, validation, investigation, supervision, methodology. **Yu Fu:** conceptualization, investigation, funding acquisition, writing – original draft, writing – review and editing, methodology, supervision, project administration, validation.

## Ethics Statement

Ethical approval was granted by the Health Research Authority in the UK (22/WS/0164). Data were collected with electronic informed consent of participants and stored securely on the University's server. A consent statement was included on each survey's introductory page. All panellists completed the consent statement prior to completing the survey.

## Conflicts of Interest

The authors declare no conflicts of interest.

## Data Availability

The data that support the findings of this study are available on request from the corresponding author. The data are not publicly available due to privacy or ethical restrictions.
